# The Roles of Thyroid and Thyroid Hormone in Pancreas: Physiology and Pathology

**DOI:** 10.1155/2018/2861034

**Published:** 2018-06-14

**Authors:** Chaoran Chen, Zhenxing Xie, Yingbin Shen, Shu Fang Xia

**Affiliations:** ^1^Institute of Nursing and Health, College of Nursing and Health, Henan University, Kaifeng, China; ^2^School of Basic Medicine, Henan University, Jinming Avenue 475004, Henan, Kaifeng, China; ^3^Department of Food Science and Engineering, Jinan University, Guangzhou, China; ^4^Wuxi School of Medicine, Jiangnan University, Wuxi, China

## Abstract

It is widely accepted that thyroid hormones (THs), secreted from the thyroid, play important roles in energy metabolism. It is also known that THs also alter the functioning of other endocrine glands; however, their effects on pancreatic function have not yet been reviewed. One of the main functions of the pancreas is insulin secretion, which is altered in diabetes. Diabetes, therefore, could be related to thyroid dysfunction. Earlier research on this subject focused on TH regulation of pancreas function (such as insulin secretion) or on insulin function through TH-mediated increase of energy metabolism. Afterwards, epidemiological investigations and animal test research found a link between autoimmune diseases, thyroid dysfunction, and pancreas pathology; however, the underlying mechanisms remain unknown. Furthermore, recent studies have shown that THs also play important roles in pancreas development and on islet pathology, both in diabetes and in pancreatic cancer. Therefore, an overview of the effects of thyroid and THs on pancreas physiology and pathology is presented. The topics contained in this review include a summary of the relationship between autoimmune thyroid dysfunction and autoimmune pancreas lesions and the effects of THs on pancreas development and pancreas pathology (diabetes and pancreatic cancer).

## 1. Introduction

### 1.1. Thyroid Physiology

Thyroid hormones (THs) are involved in several processes, such as growth, development, reproduction, and metabolism. While THs act on almost every organ in the body, research has been focused on the central nervous system [1], the cardiovascular system [2], and the skeleton [3]. Recently, increasing prevalence of metabolic diseases (including obesity, diabetes, and hyperlipemia, among others) have reestablished the focus on thyroid hormone, since THs have the ability to improve energy metabolism in the body. TH-related studies are centered on TH effects on fat degradation, glucose oxidization, and oxidative phosphorylation acceleration, and other metabolic effects [4]. Meanwhile, thyroid hormone mimetics have been developed in order to treat obesity and diabetes. Nevertheless, a deeper knowledge of the mechanisms needs to be developed in order to understand the complex physiological effects of THs.

THs include 3,5,3′,5′-tetraiodo-L-thyronine (T4) and 3,5,3′-triiodo-L-thyronine (T3); both hormones are synthesized and secreted from the thyroid gland. THs secreted from the thyroid are stimulated by thyroid-stimulating hormone (TSH), which is secreted from the anterior pituitary gland. TSH is again regulated by the thyrotropin-releasing hormone (TRH), which is produced from the hypothalamus [5]. Most of blood T3 and T4 is found in their protein-combined forms, while small amounts are found in their free form. Only free T3 and free T4 have biological action; T3 has the most potent physiological function. Free T3 largely derives from T4 via 5′-deiodinases (D1 and D2), and T3 conversion from T4 takes place inside TH target cells. D3 inactivates both T4 and T3 molecules in order to terminate thyroid hormone action in a timely manner [6]. Before being recognized by their receptors, THs must be transported into target cells by special transporters. One highly specific transporter is monocarboxylate transporter 8 (MCT8); its inactivating mutations could be the cause of diseases characterized by local TH shortage, such as Allan-Herndon-Dudley syndrome, a disorder characterized by normal TSH level and elevated T3 and decreased T4 serum levels [7]. Other TH secondary transporters include the aromatic amino acid transporter MCT10, the organic anion transporting polypeptide transporters (e.g., OATP1C1, OATP1A2, and OPTP1A4), the large neutral amino acid transporters (LAT1 and LAT2), and another amino acid transporter, the L-cystine and L-glutamate exchanger. In different organs, different expression patterns for both primary and secondary TH transporters have been described [8], suggesting that THs have different local actions in different organs.

The physiological function of thyroid hormones requires the interaction of THs and their nuclear receptors (TRs). There are two major TR isoforms, encoded on separate genes [9, 10]: TR*α* and TR*β*. The TR*β* gene encodes three TR*β* isoforms: TR*β*1, TR*β*2, and TR*β*3. All TR*β* isoforms bind to their cognate ligand T3 with high affinity to mediate target gene expression. In contrast, among the three TR*α* isoforms, only TR*α*1 is able to bind to T3 in order to activate or repress target genes, whereas TR*α*2 and TR*α*3 do not bind T3, antagonizing T3 action. TRs can bind to specific *cis* elements called thyroid hormone response elements (TREs), which are located in the promoter of target genes and form homodimers or heterodimers with retinoid X receptor (RXR) [11] and other receptors (such as estrogen receptor) [12]. Besides, THs produce nongenomic effects, which are not dependent on nuclear TRs. These effects have no structure-function relationships with THs analogs, and they have a fast onset of action by inducing membrane-related signaling pathways. The nongenomic effects are diverse; usually, they involve kinases or calmodulin, Ca^2+^-ATPase, adenylate cyclase, and glucose transporters (GLUT) [13]. Nevertheless, most T3 effects are assumed to be mediated by the regulation of TR target gene transcriptions in the nucleus.

It is well known that THs can affect the action of other hormones (such as retinoid by RXR) and also have effects on other endocrine glands. One of these glands is the pancreas, which is involved in chronic and prevalent diseases, such as diabetes. Therefore, thyroid dysfunction, including autoimmune thyroid diseases, hypothyroidism and hyperthyroidism, and abnormal TH signaling pathway, could cause pancreas dysfunctions. Sequentially, thyroid dysfunction could cause system metabolism dysfunctions, which complicate diagnoses and even affect subsequent treatments. A better understanding of the mechanisms governing metabolic diseases which considers the involvement of thyroid and THs could begin the development of a new way of tracking pancreas diseases.

### 1.2. Pancreas: Structure and Function

Pancreas is mainly made of two functional parts: the exocrine portion and the endocrine portion. The exocrine portion secretes digestive enzymes into the duodenum. These enzymes, which include trypsin, chymotrypsin, amylase, and lipase, participate in food digestion in the intestine. Trypsin and chymotrypsin digest food proteins, while amylase and lipase break down carbohydrates and fats, respectively. The endocrine portion (also known as islets of Langerhans), on the other hand, accounts for a small fraction of the total pancreatic mass. But it produces and secretes five hormones [14]: *α* cells secrete glucagon, *β* cells secrete insulin, *δ* cells secrete somatostatin, *γ* cells secrete pancreatic polypeptide, and *ε* cells secrete ghrelin. Glucagon and insulin are the main hormones responsible for the maintenance of glucose homeostasis. Since the cells of both pancreatic portions (exocrine and endocrine) originate from the same progenitor cells [15], it is possible that cell transdifferentiation occurs between the two parts.

Considering the important role of pancreas in the maintenance of normal physiological function, pancreatic dysfunction, either exocrine or endocrine, causes different diseases of pancreas, such as pancreatitis, diabetes, and pancreatic cancer. The exocrine portion corresponds to 98–99% of pancreatic mass; pancreatitis and pancreatic cancer are common diseases originating in this portion. Pancreatitis is characterized by digestive enzymes damaging pancreas structure and tissue, which induces inflammation. This condition is often caused by structural blockage, such as gallstones [16] or damage by alcohol consumption [17]. Detection of some digestive enzymes in blood, such as amylase and lipase, is often used to indicate pancreatitis. In addition, a recent study showed that pancreatic exocrine insufficiency developed after acute pancreatitis was followed by prediabetes or diabetes mellitus [18]. This suggests that pancreatitis on the exocrine portion increases the risk of pancreatic endocrine diseases. Other pancreatic disorders are pancreatic cancers, which are very difficult to cure. These tumors are usually diagnosed at a stage in which surgical treatment is no longer an effective option. Pancreatic cancers are mostly found in the exocrine part and are nowadays assumed to be the result of pancreatitis [19]. Among the different types of pancreatic cancer, pancreatic ductal adenocarcinoma represents approximately 85% of all pancreatic cancers [20]. Their pathological mechanisms are still unknown.

The endocrine portion of the pancreas is the origin of other disorders. *β* cell function disorders have attracted the attention of researchers, since they disrupt insulin secretion. As a consequence, in diabetes, glucose homeostasis is broken and tissue energy metabolism is affected. Type 1 diabetes is divided into type 1A (autoimmune) diabetes and type 1B (idiopathic) diabetes [21]. Type 1A diabetes, an autoimmune disease, corresponds to 70–90% of patients with type 1 disease. The mechanism of type 1B diabetes remains unknown. In contrast, type 2 diabetes makes up about 90% of cases of diabetes (392 million people were diagnosed in 2015) [22]. Type 2 diabetes is a complex disorder; its underlying mechanism is mostly related to defects in insulin secretion and action. Prevalence of type 2 diabetes is the cause of great social cost, since its treatment involves the use of insulin supplement and oral antidiabetic drugs and the management of complications that may arise. Considering the aforementioned pancreatic diseases, it is essential to accurately elucidate the intrinsic mechanisms that are responsible for the compromise of pancreatic function. Recent research progress has shown that the relationship between thyroid and pancreas should be addressed in order to unmask the pancreatic physiology and pathological mechanisms.

## 2. Thyroid Hormone Receptors and Transporters in Pancreas

Although the effects of THs in the development of many endocrine glands have been widely explored, knowledge about the role of THs in pancreas development has improved slowly. Since the TH receptor expression pattern has not yet been deeply examined, it was important to verify the expressions of TR mRNA and protein in the pancreas in order to provide direct evidence for possible action of THs in the pancreas. In the embryonic pancreas, the expression pattern of TRs has only been examined in animal models, both *in vivo* and *in vitro*. Recently, Aiello et al. [23] found that TR*α*1 and TR*β*1 mRNAs were expressed differently at different phases of embryonic murine pancreas development: TR*α* expression began at E12.5 (12.5 days of embryonic development) and increased steadily until reaching maximum levels at birth. In contrast, TR*β* expression was measurable from E15.5 and then rose dramatically in late development stages (E17.5) and at birth. This differential TR expression suggests the existence of different THs signaling pathways for embryonic pancreas development. However, the difference of TR expression among cell populations of the embryonic pancreas has not been explored. Besides, there is need to gather more data about TR expression in the human embryonic pancreas.

In the postnatal pancreas, an investigation about TR distribution was carried out by Lee et al. [24]. They examined the role of THs in the postnatal rat pancreas by documenting binding of the nuclear receptor to THs. Their results showed that the rat pancreas is a tissue target for THs; also, they discovered that T4 has stronger binding capacity with TRs in the adult pancreas compared to the pancreas of less than 5-day-old pups. This study did not investigate what TR subtypes were present. Afterwards, Shahrara et al. [25] demonstrated that different subtypes of TR*α* and TR*β* mRNAs (*α*1: 4.9 kb; *α*2: 3.2 kb, and 5.7 kb; *β*: 8 kb) were expressed in the human pancreas. However, TR proteins expression was not analyzed. Zinke et al. [26] further found that mRNA and proteins of TR*α*1 and TR*β*1 were detected in islets, while no expression was found in the exocrine pancreas. Also, a relative higher level of TR*α*1 protein was observed in murine pancreas *α* cells. However, TR*α*1 did not regulate mRNA glucagon levels, nor did it regulate glucagon promoter activity in a glucagon-producing cell line test. These findings suggest that TR*α* and TR*β* may play different roles in the regulation of pancreas development and pancreas function.

THs transmembrane transporters are key regulators of TH availability in target cells. This regulation is based on TH levels, since the local availability plays a role in the target organ. Therefore, it is necessary to review research done on TH transporters. Arjona et al. [27] established that monocarboxylate transporter MCT8, highly expressed in adult zebrafish, is predominantly responsible for pancreas T3 uptaking, which suggests a key role in keeping pancreas normal function. Kirat and Kato studied cellular localization and expression of MCT1–MCT5, MCT8, MCT13, and MCT14 in mRNA and protein forms in cattle pancreas [28]. In the last two years, other TH transporters have been found in pancreases, including LAT1 and MCT10 in chicken [29], and high transcript levels of OPTP1B3, 2B1, and 1A2 in adult human islets [30]. However, the efficiency of these TH transporters was not investigated.

On the other hand, TRH and TSH receptors have also been found to be distributed in the pancreas; however, no further research about their physiological effects was done [31, 32]. The studies about TRH and TSH receptors on the pancreas have found the following information: first, TRH can be secreted by the pancreas and induce the activation of epidermal growth factor (EGF) receptor by interacting with its receptor. Second, Luo and Yano demonstrated that TRH has an antiapoptotic effect on the pancreas [33]. Third, TRH has a rhythmic secretion during the day-night cycle in noncentral nervous system tissues (such liver) regulating metabolic function; however, there seems to be confusion about no rhythmic secretion of TRH in pancreas. More investigations are needed in order to reveal the detailed effect of TRH on the pancreas [34]. About TSH, there is only one report that shows that TSH can stimulate GLUT2 gene transcription by activating the p38 MAPK signal pathway [35].

## 3. Findings about the Physiological Effect of the Link between Thyroid and Pancreas

Research about the physiological role of THs in the pancreas began early. Houssay [36] first discovered that THs treatment could produce a double effect on pancreatectomized dogs in short and long periods: reversible hyperglycemia (prethyroid diabetes) or diabetes (pancreas lesions and irreversible pathogen). Thus, this study demonstrated the existence of a relationship between THs and pancreas. Another study, made by Cortizo et al. [37], found that T4 could be deiodinased to T3 in the pancreas, which promoted insulin release when the blood glucose concentration was between 2 and 8 mmol/L. However, further studies developed in the subsequent decade about THs' roles on the pancreas have focused on energy metabolism and THs' effect on insulin signaling pathways in organs other than the pancreas. These studies associated TH levels and their downstream signaling pathways with the risk of developing diabetes [38]. Recently, more research regarding the role of THs in the pancreas has been carried out, which has increased the understanding of TH physiological function in the mechanisms governing the pathology of diabetes. In the next sections, we will focus on recent research advancements about the thyroid's role in pancreas development, autoimmune pancreas disease, diabetes, and cancer.

## 4. Thyroid Hormone and Pancreas Development

### 4.1. Effects of THs on Pancreas Development in the Fetus

**Figure 1:**
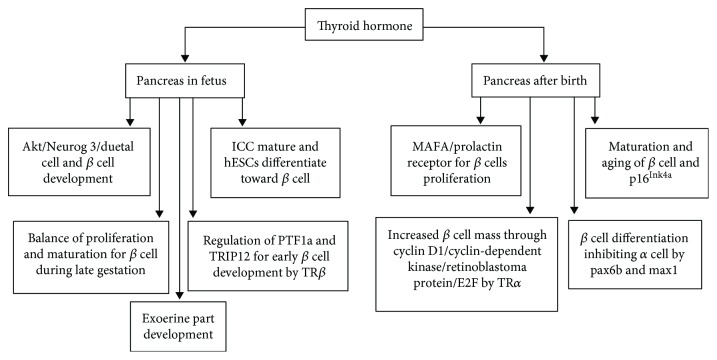
The physiological effect of thyroid hormones on pancreatic development. Akt: RAC-alpha serine/threonine-protein kinase; E2F: E2F transcription factor; hESC: human embryonic stem cell; ICC: islet-like cluster; MAFA: musculoaponeurotic fibrosarcoma oncogene family A; MNX1: motor neuron and pancreas homeobox 1; pax6b: paired box protein 6b; p16^Ink4a^: cyclin-dependent kinase inhibitor 2; PTF1a: pancreas transcription factor 1a; TR: thyroid hormone receptor; TRIP12: thyroid hormone receptor-interacting protein12.

Pancreas normal development (particularly the endocrine portion) is essential for normal body development. Alterations on pancreas functioning could begin at a fetal stage (Figure 1). For example, insulin is a potent growth factor; its deficiency is associated with fetus growth retardation in human and animal cases [39, 40]. THs are the most powerful developmental hormones. Both *in vivo* and *in vitro* studies have confirmed that THs have a role in the physiological development of the pancreas in different phases. An ex vivo culture model of embryonic murine pancreas showed that T3 induced an increase in ductal cell number, increase in mRNA levels of the proendocrine gene Neurog 3, and increase in *β* cell number. Also, T3 induced the decrease of the acinar compartment, which suggests endocrine cell transdifferentiation, possibly related to the activated Akt signaling pathway [23]. A recent study [41] showed that T3 also promotes human embryonic stem cell (hESC) differentiation into *β* cells and drives the maturation of the human fetal islet-like cluster (ICC). In both cases, insulin content and secretion were increased. It has also been demonstrated that the musculoaponeurotic fibrosarcoma oncogene family A (MAFA) is involved in this process. In contrast, TH deficiency impairs pancreas cell maturation. One study [42] showed that acute hypothyroidism impairs human C-peptide secretion at 16 weeks after transplant of pancreatic progenitors. The grafts in hypothyroid mice contained fewer *β* cell number, heterogeneous MAFA expressions, and increased glucagon cells compared to grafts from normal animals. Furthermore, the damage of pancreas function in the fetus due to maternal hypothyroid could have effects afterbirth, which could worsen with age. A study suggests it could contribute to increased risk of type 2 diabetes [43]. Gholami et al. recently revealed that lower glucose transporters (GLUTs) and glucokinase (GcK) expression in islets are a potential mechanism for higher diabetes risk in the young offspring of mothers suffering from hypothyroidism [44].

On the other hand, there have been inconsistent reports regarding the effects of T3 on embryonic pancreas development, especially in later gestation stages (Figure 1). Over the perinatal period, T3 is important for *β* cell maturation according to gestational age. In this process, *β* cells become more responsive to glucose with increased T3 levels [45–47]. However, a previous study [48] showed that normal physiological development of the fetal pancreas involves a decrease in the number of islet cells in the human fetal pancreas from gestation week 12 to gestation week 41. This latter result could be explained by another study [49], in which higher gene and protein expressions of D3, an enzyme that degrades T3, were observed in the endocrine pancreas of mice at E17.5. In these mice, D3 protein was coexpressed in insulin-positive cells. However, the question about how T3 is correctly inactivated in order to keep the concentration needed for normal pancreas development in late gestation remains. Meanwhile, neonatal mice with targeted disruption of the D3 gene (D3KO) exhibited smaller pancreatic islets, reduced absolute *β* cell mass, and lower insulin content, which seemed to be related to higher local THs levels. The mechanisms for these processes remain elusive. Recently, a striking study [50] showed that in the late fetal sheep pancreas, decreased thyroid hormone levels due to thyroidectomy could induce an increased *β* cell mass. In this study, absolute and relative *α* cell mass during late gestation remained unchanged. This research was inconsistent with the results discussed above, in which T3 had been shown to stimulate proliferation and insulin secretion in pancreatic *β* cells during gestation. All the above results hint at the differences existing between pancreas *β* cells at proliferation and mature stages. Thus, low local TH concentration in pregnant women in late pregnancy stages may only maintain proliferation of pancreatic *β* cells and insulin basal secretion, while it does not promote the maturation of glucose-stimulated insulin secretion regulation ability. This would lead to less pancreas sensitivity to glucose stimulation, deteriorated pancreatic islet morphology, and less insulin secretion. These effects could be contributing to the development of diabetes regardless of adult euthyroid offspring [51]. Therefore, the results of the above-mentioned study could indicate that maternal hypothyroidism causes *β* cell maturation and reduces *β* cell number in the fetus. Physiological T3 levels are supposed to promote *β* cell maturation and glucose-stimulated insulin secretion ability from immature islets at late pregnancy stages. However, mRNA and protein expression of MAFA was not investigated. This is rather puzzling, since MAFA has been found to exhibit double effects: It promotes *β* cell maturation and proliferation through its target genes after birth [52]. While these findings are key in the understanding of the mechanisms governing diabetes at late life due to failures in programmed development of the pancreas resulting from maternal thyroid function status, the processes and molecular mechanisms of high-level THs and low-level THs procedurally regulating pancreas development are still elusive. To date, two possible reasons for the phasic different action of THs on pancreas development in the whole fetus period have been identified: firstly, different expressions for TH signaling pathways. However, these mechanisms have not yet been completely elucidated, including TH receptor, transporter, cell cycle, and mature signal molecules. For example, a possible molecular signal was described in a recent research by Rouintan et al. [53]. In this research, nitric oxide overproduction through high nitric oxide synthase (NOS) activity increased plasma glucose levels and decreased insulin secretion in fetal hypothyroid rats delivered from 6-propyl-2-thiouracil-treated pregnant rats. The underlying mechanism, which was not fully described, may be related to different steps of the insulin secretion pathway, such as decreased activities of both hexokinase and glucokinase, not related to the insulin pool of *β* cells [54]. Still, there is still need for further research in order to fully understand this pathology. Regarding TR, there is only one report [55] showing that the TR number and positioning changed in pancreatic *β* cell of postnatal rats, which will be discussed below. A recent study [56] showed that thyroid hormone receptor-interacting protein (TRIP) 12, an E3 ubiquitin-protein ligase, is a recently discovered component of pancreas transcription factor 1a (PTF1a), which plays a crucial role in the pancreas early development and in the maintenance of the acinar cell phenotype. TRIP12 can play a role in PTF1a downregulation induced by T3, which suggests that TR*β* is unfavorable for pancreas endocrine development. Secondly, other endocrine hormones, such as leptin, that have both inhibitory and stimulatory effects [57, 58], were found to be influenced by THs and could indirectly alter pancreas development, even inducing the opposite effect. Therefore, appropriate TH concentrations in different fetus phases are needed for normal development of the pancreas. The dysfunction of THs may be the culprit of diabetes pathogenesis.

### 4.2. Effects of THs on Pancreas Development after Birth and in Adult

Although the effects of THs on pancreas development in neonatal period are less crucial than in fetal life, pancreas function is still considered functionally immature in the neonatal phase (Figure 1). Meanwhile, *β* cells lack glucose responsiveness in neonatal rodents under shortage of THs. For example, AvRuskin and Juan [59] reported a 30-year-old Hispanic male who showed transient neonatal diabetes mellitus at 4 months and was diagnosed with hypothyroidism with positive thyroperoxidase antibodies. This case argues in favor of an association between hypothyroidism and immature pancreas function with diabetes occurring after birth; however, several surveys and studies about the effect of THs on human postnatal pancreas development are still less illuminating. In animal studies, a strong amount of data has confirmed that THs are essential for pancreas development. Lee et al. [60] found postnatal developmental retardation of the rat pup pancreas after 6-n-propyl-2-thiouracil treatment. He also proved that the effects of THs on pancreas development included both the endocrine part and the exocrine part. In the case of pancreas exocrine function, a delayed induction of pancreatic *α*-amylolytic activity was found in sucklings fed by thyroidectomized mothers [61]. Regarding the pancreas endocrine part, hypothyroid lactating rat mothers could bring about multiple pathology results: higher plasma glucose in glucose tolerance test, higher insulin resistance, and reduced islet diameter in their adult male offspring [62]. However, the effect of THs on the postnatal development of the endocrine pancreas is complicated. It possibly involves several levels of signal pathways [55]. First, the TH signaling pathway may be enhanced to some extent due to decreased D3 expression and increased D1 levels. D1 levels increase in islet, which promotes higher levels of active T3 in one-month-old rats. Changes in TH-predominant receptor isoforms in insulin-positive cells throughout postnatal development provide the second regulation mechanism of TH action on the pancreas. TR*α* is predominant at early ages, then TR*α* and TR*β* are equal in postnatal days 9 to 15, and afterwards TR*β* is predominant at the mRNA level. Regarding protein levels, nuclear TR*α* protein synchronizes with its mRNA expressions. TR*α* protein is lower in levels, and it is less localized in the nucleus, while TR*β* protein localization changes from cytoplasm to nucleus. Nevertheless, the reason for this differential shift of the two TR isoforms after birth is unclear. Moreover, T3 treatment increased protein expressions of nuclear TR*α* and (more notably) cytoplasmic TR*β* within one month after birth. This suggests a possible positive feedback on TRs by THs. This study also showed that MAFA is a specific target of T3-mediated postnatal effects, such as higher glucose-induced insulin secretion and better functional maturation of *β* cells. These results match those found in the fetus, in which TRs directly interact with two putative TREs in the MAFA gene. In addition, MAFA could promote *β* cell proliferation through prolactin receptor activation and downstream signaling [52]. Another study [63] demonstrated that the higher expressions of TR*α* play an important role in *β* cell replication and in the expansion of the *β* cell mass in rat pancreatic *β* cell lines (RIN5F). This was proved with the use of a recombinant adenovirus vector, AdTR (*α*). The enhanced cyclin D1/cyclin-dependent kinase/retinoblastoma protein/E2F pathway was found to be involved in the process. This result is obviously inconsistent with the TR-changing mode in development. The real effect of T3 may be more complicated than anticipated; the underlying mechanism needs further exploration. In addition, the effect of THs on pancreas development could be the consequence of differently activated differentiation genes of *α* cells and *β* cells. Matsuda et al. [64] found that exogenous THs precociously promote *β* cell differentiation by upregulating pax6b and mnx1 genes, while THs also inhibit *α* cell development and function by downregulating arxa during the zebrafish larval-to-juvenile transition. This is done in order to maintain glucose homeostasis. However, the mechanism was not discussed in the study; therefore, there are more signaling pathways beyond TR waiting to be discovered.

From the above-cited studies, it can be stated that the mechanisms governing the effect of THs on pancreas development in the postnatal period are just as elusive as those on the in fetus. The latest study [65] on the topic showed that T3 could induce maturation and aging of pancreatic *β* cells. Also, p16^Ink4a^ (a *β* cell senescence marker and effector) mRNA expression was increased in rodent and human *β* cells after T3 treatment. The findings done in this study indicated the other striking extreme effect of THs in pancreas development when compared with previous understanding that TH deficiency in prenatal, postnatal, or even infancy was closely related with glucose intolerance and reduced glucose-stimulated insulin secretion contributing to diabetes development in adulthood.

## 5. Roles of Thyroid and Thyroid Hormone in Pancreas Pathogenesis of Diabetes

Previous studies declare that the pathogenesis of type 1 diabetes resides on autoimmune destruction of pancreatic *β* cells. This destruction results on chronic inflammatory infiltrating, which makes it impossible for the pancreas to produce enough insulin for normal functioning [66]. The only therapeutic option for type 1 diabetes is to replace insulin. Considering this, the knowledge of more mechanisms of type 1 diabetes may offer new insights into methods for diabetes prevention. Recent studies show that autoimmune thyroid diseases often appeared simultaneously with type 1 diabetes, which suggests that thyroid malfunction may be involved in the pathology process of diabetes. Autoimmune thyroid diseases present as hypothyroidism or hyperthyroidism, mainly depending on different types of plasma autoantibodies: hypothyroidism is often accompanied by thyroid peroxidase antibodies (TPO Abs) or thyroglobulin antibodies (Tg Abs); hyperthyroidism is often associated with the presence of thyroid-stimulating hormone receptor (TSH) antibodies.

### 5.1. Effects of Autoimmune Hypothyroidism on Pancreas Dysfunction

Autoimmune hypothyroidism has also been found to alter pancreas function related to diabetes. Epidemiological studies have shown that autoimmune pathologies against the thyroid gland, such as TPO Ab and Tg Ab (which cause hypothyroidism), often coexist with autoimmune pancreas disorders with islet lesions in cases of diagnosed type 1 diabetes. In fact, one study [67] found that a patient with type 1 diabetes with autoimmune thyroid disease had also higher than normal levels of characteristic markers of autoimmune disorders, such as glutamic acid decarboxylase autoantibody (GAD Ab) and insulinoma-associated protein 2 autoantibody (IA-2 Ab). These markers are important markers of pancreas pathological damage; they last longer and are found at higher titers than in patients with only type 1 diabetes. Using a regression analysis, Murao et al. [68] demonstrated that plasma TPO Ab and IA-2 autoantibody levels, as well as low plasma C-peptide levels under fasting conditions, were closely related to the need of insulin treatment for type 1 diabetes patients. Thus, these Ab are an indication of the beginning of pancreas islet damage. In another study, levels of autoimmune pancreas marker (GAD Ab) and insulinoma-associated antigen 2 were considerably higher in patients with autoimmune thyroid disease and diabetes than in only autoimmune thyroid disease [69], and they were closely associated with increased prevalence of diabetes mellitus. In patients with autoimmune thyroid disease, the presence of GAD Ab was shown to be consistent with the onset of diabetes, lower body mass index, higher hemoglobin A1c level, and higher frequency of insulin therapy at a younger age. However, a recent survey [70] showed that less than twenty percentage of Asian Indian female patients suffering from hypothyroidism presented islet cell Ab with normal GAD Ab. Prevalence of islet antibody in patients with autoimmune thyroid was also low. However, in patients with long-term primary hypothyroidism, Langerhans cell histiocytosis did promote the onset of diabetes [71, 72]. In autoimmune thyroid diseases, such as Hashimoto's thyroiditis, the thyroid is underactive and it does not produce enough thyroid hormones. This directly affects the pancreas through TRs.

It has been accepted for a long time that the decrease in thyroid hormone levels and its function is associated with diabetes and its corresponding syndromes [73]. Pancreatic dysfunction is an important marker for diabetes, regardless of types. Therefore, it is important to review whether lower thyroid hormone levels due to something other than thyroid dysfunction are also a reason for pancreas disorder. So far, only few human reports of pancreas dysfunction in primary hypothyroidism patients without autoimmune thyroid disease have been made. In these cases, patients often presented with multiple endocrine failures, such as liver, spleen, and adrenal gland, besides pancreatic malfunction [74]. In addition, Handisurya et al. [75] evaluated *β* cell function and insulin sensitivity in subjects with both overt and subclinical hypothyroidism, as well as the effects of T4 replacement therapy. Their findings were unexpected, since they demonstrated that glucose-induced insulin secretion and proinsulin levels in hypothyroidism subjects were increased, despite their decreased basal plasma insulin levels. This result may be due to the existence of an overload of islet working; however, pancreatic pathologies were not explored. Meanwhile, clinical data about pancreas function in hypothyroidism patients is lacking.

In contrast to the previously described study, most research has been concentrated on hypothyroidism in animal models. Results from those studies have not been completely consistent with clinical results. These types of research were initially conducted in rats after thyroid hormone deprivation. *In vivo* thyroid removals on rats induced diminished insulin secretion in response to high glucose levels. It also induced *β* cell ultrastructure abnormalities: large amount of secretory granules and a marked enlargement of their clear halo. There were also signs of hyperactive granular endoplasmic reticulum [76]. Regardless of these alterations, the number, volume, and content of insulin of the pancreatic islets were not affected. Metabolic and secretory changes, as well as the ultrastructural modifications, were corrected when circulating T3 and T4 levels returned to normal values after thyroid hormone administration. Also, islet insulin secretion was positively correlated with serum T3 and T4 concentrations. In a recent study [77], propylthiouracil-induced hypothyroidism in male rats also led to impaired glucose tolerance due to reduced glucose-stimulated insulin secretion. In an *in vitro* study, Diaz et al. [78] found that the dynamics of glucose-induced insulin secretion on isolated pancreatic islets was decreased in the first and second phases of insulin output, which was reversed by T4 administration. In a female rabbit hypothyroidism model [78], while the cell number in large and medium islets was reduced, their number on small islets was increased. At the same time, TR*α*1–2 and TR*β*1 expression was upregulated, possibly as a way to compensate for reduced TH levels. Unchanged serum glucose levels may be due to the estrogen effect compared to increased serum glucose levels in the aforementioned male rat model above [31] and hypothyroidism patients. It is possible that estrogen has a protective effect on the pancreas-ameliorating impaired effect of THs on pancreas and glucose metabolism.

Regarding the study of mechanisms, an *in vivo* study demonstrated that methimazole-induced hypothyroidism promotes immune cell infiltration into pancreatic islets of female rabbits, which could be inducing pancreatitis and insulitis [79]. Other mechanisms involved in islet dysfunction may be related to different signals. Furthermore, a study showed that TH receptor disorder is closely related to pancreatic *β* cell survival after endoplasmic reticulum (ER) stress. This study found higher levels of TR*α*-activated transcription factor 4 (ATF4) and heme oxygenase 1, which facilitates adaptation to oxidative ER stress in the pancreas of HFD-treated mice [80]. In addition, it is known that glucose sensing is the initial event for glucose-stimulated insulin secretion. Glucokinase-specific activity and GLUT 2 protein expression were significantly decreased in isolated islets from hypothyroid rats, pointing out a possible mechanism for insulin secretion deficit [81]. Besides, TH deprival could decrease proinsulin gene expression and the attachment of eukaryotic translation elongation factor 1A (eEF1A) to the cytoskeleton in INS-1E cells, which is essential to attaching transcripts of proinsulin to the cytoskeleton and modulating their stability and translation rate [82]. Moreover, oxidative stress in hypothyroidism may directly cause pancreas injuries. For example, lipid peroxidation has been found to be significantly increased, while antioxidant enzyme activity was significantly decreased in hypothyroidism cases [83], along with reduced glutathione levels [84]. These results indicate that oxidative stress may play a role in the progressions of pancreatic *β* cell dysfunction, reduced *β* cell mass, and decreased glucokinase activity.

### 5.2. Effects of Autoimmune Hyperthyroidism on Pancreas Dysfunction

In contrast, reports about the effects of autoimmune hyperthyroidism on pancreas function seem to be inconsistent. A survey [85] showed a dual *β* cell defect in hyperthyroidism patients: decreased insulin release in response to hyperglycemia and increased proinsulin levels both in the fasting state and in response to a meal. Meanwhile, glucose intolerance and the accompanying increased lipid oxidation suggest quantitative and qualitative *β* cell defects. On the other hand, autoimmune hyperthyroidism seems to predict the presence of pancreas antibodies and, to a lesser degree, diabetes development. Maugendre et al. [86] found that although Graves disease (GD) patients have higher levels of islet cell Abs (ICA) compared to normal controls; they also had a lower frequency of evolution towards diabetes in the 14-year follow-up. Also, the frequency of islet cells Ab, which reflects on pancreas injury, was less associated with Graves disease than with GAD64 Ab. The elevated GAD Ab was likely related to autoimmune thyropathy, because the frequencies of GAD65 Ab and GAD67 Ab in toxic nodular goiter (a nonautoimmune form of hyperthyroidism) were lower [87] and modulations of TSH receptor antibodies level by carbimazole had no effect on the development of insulin autoantibodies [88]. All of the above suggest that, under certain circumstances, GAD65 Ab and GAD67 Ab may be a more accurate predictor of autoimmune hyperthyroidism than of nonautoimmune hyperthyroidism; therefore, the subject deserves further investigation.

Moreover, high TH levels in hyperthyroidism, except in the case of autoimmune diseases, could also influence pancreas function. Treatment with high levels of T4 could induce pancreatic *β* cell apoptosis, as was shown by an increase in transferase-mediated deoxyuridine triphosphate-biotin nick end labeling (TUNEL) and caspase-3 expressions in rat *β* cells [89]. Recently, it was demonstrated that impaired glucose-stimulated insulin secretion and reduced *β* cell mass occurred in pancreatic islets of hyperthyroid rats [90]. The former effect may be related to decreased sensitivity of ATP-sensitive K^+^ (K^+^(ATP)) and L-type Ca^2+^ channels of the *β* cells. Meanwhile, the decreased half-life of circulating insulin [91] in hyperthyroidism aggravates the pancreas burden and diabetes. Therefore, although there is a lack of evidence to prove that elevated THs cause diabetes, high THs do aggravate pancreas pathogenesis during diabetes.

In brief, these results suggest that autoimmune thyroid diseases or their accompanying TH abnormal levels could play an accelerating role in autoimmune pancreas collapse. Thus, more attention should be paid to the status of the pancreas under hypothyroidism or hyperthyroidism in order to understand the mechanism of pancreas dysfunction in cases of insufficient or excessive THs and/or action. It seems that higher levels of THs could aggravate pancreas dysfunction. In contrast, lower thyroid hormone levels may indicate the offset of pancreas pathogenesis. Nonetheless, in the next section there is a necessary review of how THs can alter pancreas function in order to deeply understand the mechanisms of THs actions in pancreas pathology.

### 5.3. Possible Molecular Mechanisms between Thyroid and Pancreas Pathologies

As stated above, the connection between autoimmune thyroid and autoimmune pancreas dysfunction is essential to understanding the intrinsic mechanisms that cause pathologies on these organs. So far, the mechanisms linking autoimmune thyroid and autoimmune pancreas disorders have been seldom explored. Knowledge is limited to some relevant molecules that seem to connect thyroid and pancreas pathologies. First, it is well known that GAD exists in the pancreas, where it plays an important role in pancreas normal function. Interestingly, GABA, the product of GAD-processed glutamic acid, is also found in the thyroid [92]. The roles of GABA and GAD in the interrelationship between the thyroid and pancreas need further research, since they may interpret autoimmune diseases occurring in both organs. Another key molecule that could be linking thyroid and pancreas is transcription factor Gli-similar 3 (GLIS3). Variations of GLIS3 are strongly associated with both type 1 and type 2 diabetes in some populations [93]. GLIS3 can also coordinate with pancreatic and duodenal homeobox 1 (PDX1), MAFA, and neurogenic differentiation factor 1 (an insulin synthesis-related transcription factor) to potently regulate insulin gene transcription. A case [94] of neonatal diabetes mellitus and congenital hypothyroidism revealed transcription factor GLIS3 mutations, which is expressed in both pancreas islets at an early developmental stage and in the thyroid. This molecule may offer a new light in order to explain a possible connection between pancreas and thyroid dysfunction, because insulin 2 promoter-driven Cre-mediated GLIS3 deletions to a different extent is negatively correlated with expression levels of insulin. However, variations of GLIS3 in hypothyroidism and pancreas dysfunction need further exploration. A third molecular candidate for connection is cytotoxic T-lymphocyte-associated protein 4 (CTLA4) and its allelic variants, which are associated with autoimmune thyroid failure and faster *β* cell exhaustion in latent autoimmune diabetes in adults [95]. CTLA4 is a protein receptor that downregulates immune responses. Its variants may be a molecular signal that initiates autoimmune pathologies of thyroid and pancreas. Still, accurate descriptions of the mechanism by which these molecules link thyroid and pancreas pathology still await future exploration.

## 6. Thyroid Hormone Treatment Ameliorates Pancreas Dysfunction in Diabetes

**Figure 2:**
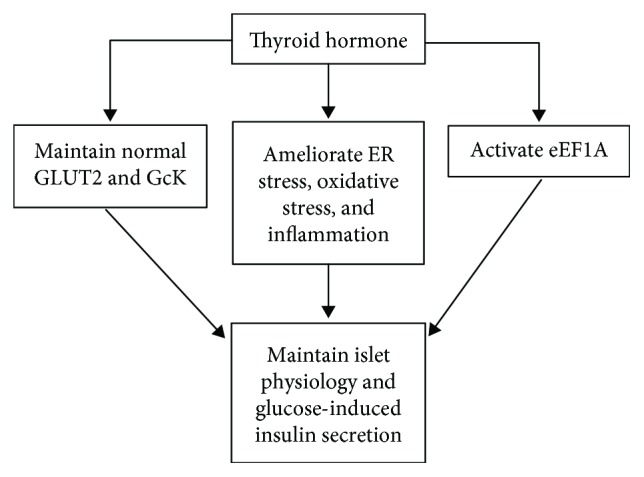
The effect of thyroid hormones on normal pancreatic physiology. ER: endoplasmic reticulum; eEF1A: eukaryotic elongation factor 1A; GcK: glucokinase; GLUT2: glucose transporter 2.

Considering that thyroid hormone dysfunction affects pancreas to different degrees and that hypothyroidism coexists with decreased pancreas function in diabetes [96], several researchers have considered TH administration to improve pancreas function *in vivo* in addition to ameliorate body glucose consumption and mitigate other diabetic syndromes (Figure 2). For type 1 diabetes treatment, a famous investigation [96] stated that T3 treatment could counteract streptozotocin-induced diabetes in mice. The islets of diabetic mice showed improved function after T3 treatment, which was seen in number, shape, dimension, consistency, ultrastructure, insulin, decreased apoptosis, and glucagon levels, besides improved glucose tolerance and elevated serum insulin levels. The mechanism by which T3 promotes its survival effect may be related to elevated phosphorylated Akt. A latest study [97] showed that levothyroxine could remarkably enhance glucose clearance and blunt the onset of experimental type 1 diabetes mellitus in mice. Interestingly, levothyroxine treatment in healthy mice remarkably increased both proliferation and apoptosis of pancreatic *β* cells with the maintenance of a highly insulin-expressing *β* cell population. In this fashion, circulating insulin levels were increased and the insulin receptor substrate 1/AKT signal was persistently activated in insulin-target tissues. Afterwards, levothyroxine-treated mice could sustain streptozotocin challenging and blunt experimental autoimmune diabetes by ensuring *β* cell proliferation and preserving insulin-expressing cells. In the case of type 2 diabetes, T3 treatment attenuated blood glucose and increased insulin sensitivity in *db/db* mice in a dose-dependent manner [98]. More importantly, higher doses of T3 could reverse insulin resistance and improve pancreas islet function, possibly by increasing the expression of the neurogenic differentiation factor. Although it was demonstrated that *in vitro* T3 could enhance insulin-induced tyrosine phosphorylation of insulin receptor substrate by TR*α*1, TR expressions in the pancreas were not addressed in this study. However, regarding human studies, so far there have been few reports connecting T3 levels to pancreatic *β* cell function in prediabetic and diabetic patients. A multiple linear regression epidemiologic study [99] showed that free T3 is an independent variable with a positive correlation with all indexes of *β* cell function in the prediabetes and euthyroid group. In another study [100], a total of 266 patients with type 2 diabetes and normal thyroid function were recruited in order to study the associations between serum T3, FT3, and acute insulin response (AIR) in type 2 diabetes. The result showed a positive correlation between T3, FT3, and acute insulin response (AIR), suggesting that serum T3 and FT3 may be the independent risk factors for predicting islet *β* cell function in type 2 diabetics. Nevertheless, it remains unknown whether normal physiological concentrations of T3 and FT3 are necessary for islet *β* cell functions among type 2 diabetics. In addition, p43, a mitochondrial T3 receptor, was found to have an important effect in pancreas function. p43 is a mitochondrial transcription factor found in this organelle genome; it stimulates mitochondrial biogenesis, and its knockout causes defects of insulin secretion and decreases pancreatic islet density both *in vivo* and in isolated pancreatic islets. It also causes loss of glucose-stimulated insulin secretion [101]. Mice that specifically lack p43 could be experimenting downregulation of the expressions of the GLUT 2 and Kir6.2, which are a key components of the K^+^ (ATP) channel for insulin excreting. However, in cellular signaling molecular mechanisms, some molecules may be involved in corrective effect on TH function. In this scenario, T3 surplus plays negative roles. For example, peroxisome proliferator-activated receptor (PPAR) *α* competed with T3 regulating islet adaptations to starvation and dietary lipid-induced insulin resistance. Its activation could restore the decrease in glucose-stimulated insulin secretion (GSIS) that occurs on hyperthyroidism [102]. Unfortunately, the reason behind hyperthyroidism-induced pancreas dysfunction has not yet been clarified.

In contrast, other studies were carried out *in vitro* in order to explore the mechanism of TH action on the pancreas. Verga Falzacappa et al. [103] firstly established that T3 has an antiapoptotic effect on cultured *β* cells that underwent apoptosis. T3 also promoted cell proliferation in islet *β* cell lines (rRINm5F and hCM), leading to increments of cell number and cell viability, and regulating cell cycle-related molecules (cyc A, D1, E, and p27). The antiapoptotic mechanism related to T3 was found to be PI3K dependent. In an ex vivo islet cell culture, which is often used as a good strategy of islet transplantation, T3 treatment expands islet cell mass and elevate islet viability [104]. These results coincide with those found in the former *in vivo* study. Secondly, Falzacappa found that a TH receptor subtype contributed to the different effects of TH on pancreas islets. Coimmunoprecipitation and colocalization experiments [105] revealed that TR*β*1 and the PI3K p85*α* subunit were able to form a complex at the cytoplasmic level in both rRINm5F and hCM cell lines. In these cell lines, nongenomic pathways are involved in mitogenesis, survival, and differentiation. Simultaneously, TR*β* also promoted the proliferation of pancreatic exocrine acinar cells. However, the role that TR*α* seemed to play is more important, since they have a role in transforming elastase-expressing pancreatic acinar cells into synthesized insulin-producing cells. T3 treatment could increase the associating between TR*α* and the p85*α* subunit of PI3K, which would result in the phosphorylation and activation of Akt, as well as increased expression of Pdx1, Ngn3, and MAFA in purified acinar cells. Due to these effects, excessive TR*α* expressions promoted the transformation of pancreatic acinar cells into insulin-producing cells [106], related to *β* cell regeneration. A previous report [107] also showed similar results (even if they did not discuss TR): T3 induced cell cycle perturbations and played an important role in the transdifferentiation of a human pancreatic duct line (hPANC-1) into pancreatic-*β* cell-like cells, together with an increase in GLUT 2 mRNA levels and a decrease of the ductal differentiation marker cytokeratin 19. At the subreceptor level, TRIP3 was shown to alter glucose metabolism by enhancing the transcription activity of the hepatocyte nuclear factor-4*α* (HNF-4*α*) [108]. In this process, HNF-4*α* gene mutation in pancreas MIN6 cells was associated with a subtype of maturity-onset diabetes of the young (MODY1) that was characterized by impaired insulin secretion in response to high glucose load. As a whole, THs can have a physiologically stimulatory effect *in vitro* on cellular growth. The related mechanism of cellular signaling is possibly related to the mitogen-activated protein kinase/extracellular regulated kinase (MAPK/ERK) pathway connected to TR*β*, according to a study in INS-1 cells [109].

## 7. Thyroid Hormone and Pancreatic Cancer

Pancreatic cancer mainly starts within the part of the pancreas which makes digestive enzymes and is dominated by pancreatic adenocarcinoma which is by far the most common type. As an important risk factor, chronic pancreatitis appears to almost triple risk, and new-onset pancreatitis may be a symptom of a tumor together with diabetes [110]. Meanwhile, a close association between autoimmune hypothyroidism and autoimmune pancreatitis also indicated that thyroid dysfunction could increase the risk of pancreas cancer. Moreover, in further data of pancreatic adenocarcinoma patients, autoimmune thyroid disease with antithyroid autoantibodies was more closely associated with pancreatic cancer because circulating TH levels were normal [111]. But the cause-and-effect relationship is still elusive because pancreatic cancer could possibly cause impaired immunoregulation, that is, generate thyroid autoantibodies. Although increased proliferation of pancreatic acinar cells could be measured by the method of BrdU incorporation, the effects of THs on pancreatic cancer showed to be bidirectional by now according to the reports and the mechanism had been just partly explored. On the one hand, T3 could induce cell proliferation and metabolism in the insulinoma cell line hCM by activating the TR*β*1/Akt pathway [112]. In addition, upregulated integrin *α*V*β*3, as a cell surface receptor for T4, may play a vital role in the development of pancreatic cancer with lymph node metastases. When a specific blocker for *α*V*β*3 is used, reduced PANC-1 tumor mass in a mouse xenograft model, increased proapoptotic BcLx-s expression, reduced tumor hemoglobin content (a marker of angiogenesis), and decreased expression of epidermal growth factor receptor (EGFR) could be observed [113]. A recent study [114] showed that although the prevalence of hypothyroidism in patients with pancreatic cancer was 14.1%, exogenous thyroid hormone treatment in pancreatic cancer cell lines also significantly increased cell proliferation, migration, and invasion, suggesting that exogenous thyroid hormone may contribute to the progression of pancreatic cancer. On the other hand, the tumor-suppressive and antiangiogenic effects of THs were also reported. The effect of T3-dependent cell growth inhibition was demonstrated by fluorescent-activated cell sorting analysis and by cell cycle-related molecule analysis. In this study [115], synergic inhibiting effects of T3 and chemotherapy on ductal pancreatic adenocarcinoma proliferation pointed another pathway to treat pancreatic adenocarcinoma *in vitro*. But the concentration and mechanism were not discussed in the study. A similar report that thyroid hormone inhibits the growth of pancreatic cancer xenograft in nude mice may be involved in more complex mechanism such as the effect of TR*β* in inhibition of tumorigenesis [116]. Therefore, there is a long way to decipher the mechanism.

## 8. Conclusion and Future Perspectives

Thyroid function has been associated with pancreas function since the phenomenon that THs improved elevated blood glucose levels in pancreatectomized animals was observed [32]. The identification of functional thyroid receptors in the pancreas gave more insights into the role of thyroid hormone in the pancreas. In this review, the relationship between autoimmune thyroid and autoimmune pancreas has been summarized for the first time. Also, a few molecules that may initiate the connection, such as GAD, GLIS3, and CTLA4, were explored. However, there still remains lack of important data about the connections between thyroid and pancreas. Therefore, whether the thyroid or the pancreas is the first to develop the pathology remains unknown, as do the exact mechanisms. In addition, thyroiditis could increase the risk of pancreatic cancer.

On the other hand, normal TH levels and normal TR expression directly concern normal pancreatic function. First, appropriate TH levels are necessary for pancreas development. Recently, Thornburg and Chattergoon [117] proposed that appropriate thyroid hormone concentration in the fetus is required for normal development of the pancreas, which opens a new area of investigation regarding THs' role in pancreas, even if the underlying mechanism remains unclear. Also, TH abnormality in adult hyperthyroidism and hypothyroidism affects pancreas endocrine function in diabetes occurrence at varying degrees. Lower TH levels or lower function pathways seem to be related to pancreas pathogenesis. Higher TH levels, on the other hand, seem to promote the development of pancreas dysfunction. In recent years, THs or its agonists were considered for the treatment of prediabetes and diabetes exhibiting hypothyroid symptoms. Appropriate thyroid hormone concentrations and signal pathways in adults are required for maintaining normal pancreas function. These pathways also required further investigation, which could provide a new dawn for the diagnoses and treatment of diabetes and related diseases.
